# A Minimal Mechanism for the Phase Transition-Driven Mpemba Effect in Systems with a Single Order Parameter

**DOI:** 10.3390/e28010100

**Published:** 2026-01-14

**Authors:** Li Li, Ji-Xuan Hou

**Affiliations:** School of Physics, Southeast University, Nanjing 211189, China

**Keywords:** Mpemba effect, phase transition, free-energy landscape, barrier-crossing kinetics

## Abstract

The Mpemba effect, where a hotter system can enter a cold phase faster than a cooler one, remains a counterintuitive phenomenon whose origins are still being unraveled. In this work, we propose and demonstrate a simple and general mechanism for the genuine, phase transition-driven Mpemba effect. Our mechanism requires only a single order parameter to describe the system’s state and operates within a standard Markovian framework, distinguishing it from previous models that necessitate multiple order parameters or non-Markovian dynamics. The core of the effect lies in the distinct relaxation pathways following a sudden quench: a system prepared at a higher initial temperature may be projected onto a region of the final free-energy landscape that requires it to cross fewer energy barriers to reach the stable low-temperature phase, whereas a system prepared at an intermediate temperature may be trapped in a metastable state, requiring the crossing of multiple barriers. We concretely illustrate this mechanism using the extended spin-1 Nagle–Kardar model, where an appropriate choice of parameters yields the requisite free-energy topography. Through extensive Monte Carlo simulations, we confirm that the initially hot system consistently reaches the final ferromagnetic phase in less time than its initially warm counterpart, thereby exhibiting a robust Mpemba effect. Our findings provide a minimal and clear explanation for how the initial state’s position in order parameter space can dictate the kinetics of a first-order phase transition, leading to this anomalous acceleration of cooling.

## 1. Introduction

The Mpemba effect—named after Tanzanian high-school student Erasto Mpemba who, together with physicist Denis Osborne, brought renewed attention to the phenomenon in the 1960s—refers to the counterintuitive observation that, under certain conditions, initially hotter water can freeze faster than cooler water when both are placed in the same subzero environment [1]. While anecdotal accounts of this effect date back to Aristotle, Francis Bacon, and René Descartes [2], its reproducibility and physical origin have remained controversial for centuries [3]. Modern research has confirmed that the effect is not unique to water and can manifest in a wide variety of physical systems [4,5,6,7,8,9,10,11], including granular fluids [12,13,14], spin glasses [15], nanotube resonators [16], and quantum systems [17,18,19,20,21,22,23,24,25,26,27,28].

Despite this breadth of experimental and theoretical realizations, a crucial conceptual distinction is often overlooked in the literature. We propose to categorize Mpemba-related phenomena into two distinct classes. The **first class**, which we term the **Mpemba-like effect**, concerns the anomalous crossing of thermal relaxation curves in the absence of any phase transition: a system prepared at a higher initial temperature relaxes to the bath temperature faster than one prepared at a lower initial temperature [11,29]. This class has been extensively studied within the framework of Markovian stochastic dynamics, where the effect arises from a nonmonotonic projection of the initial thermal state onto the slowest relaxation eigenmode of the system [29]. While mathematically elegant, this scenario lacks the defining feature of the original observation: the transition from a liquid to a solid phase.

The **second class**, which we reserve for the genuine **Mpemba effect**, explicitly involves a phase transition [30,31,32,33,34,35,36,37]. In this context, the relevant question is not simply which system cools faster, but which system **enters the low-temperature phase first**. The original experiment by Mpemba involved the freezing of an ice cream mixture, a process fundamentally governed by nucleation and phase change [1]. This distinction is critical: a system may cool rapidly yet remain in a supercooled metastable liquid state, while another, which cools more slowly in temperature, may nucleate a solid phase earlier. Consequently, the second class of the effect is intrinsically linked to the landscape of free energy, the presence of metastable states, and the kinetics of barrier crossing.

Research on this second, phase transition-driven class of the Mpemba effect remains comparatively scarce. A significant body of work from our group has focused on this very problem. In mean-field spin systems coupled to a finite thermal reservoir, we demonstrated a non-Markovian Mpemba effect where the initially hotter system heats the reservoir more, thereby reducing the free-energy barrier for the phase transition and accelerating its completion [34,35]. In parallel, we proposed a theoretical model wherein the initial condition of the hotter system lies in the basin of attraction of the stable low-temperature phase, while the cooler system is trapped in a metastable state, leading to a dramatic difference in transition times [36]. These studies highlight that the path a system takes through its order parameter space during a quench—not just its instantaneous temperature—is the decisive factor. Notably, some previous theoretical models for the phase transition-driven Mpemba effect, such as those based on the Blume–Emery–Griffiths model, require at least two coupled order parameters to create the necessary topological separation between basins of attraction [36,37].

Notwithstanding these advances, the known physical mechanisms for the phase transition-driven Mpemba effect are limited. The central open question is whether other, more general pathways can lead to this phenomenon. This paper presents a theoretical model that offers a clear mechanism for the Mpemba effect occurring during a canonical first-order phase transition. We demonstrate that the key lies in the system’s initial state relative to its final target state after quenching. Specifically, a system prepared at a higher temperature may find itself closer to the final equilibrium state in the order parameter space, requiring it to overcome fewer free-energy barriers to reach the low-temperature phase. In contrast, a system starting from an intermediate temperature might be trapped in a metastable state, necessitating the crossing of multiple barriers before reaching the same final state. This difference in the relaxation pathway directly leads to the counterintuitive observation that the initially hotter system can reach the cold phase faster.

## 2. A Novel Mechanism for the Mpemba Effect: The Role of Metastable States and Energy Barriers

**Figure 1:**
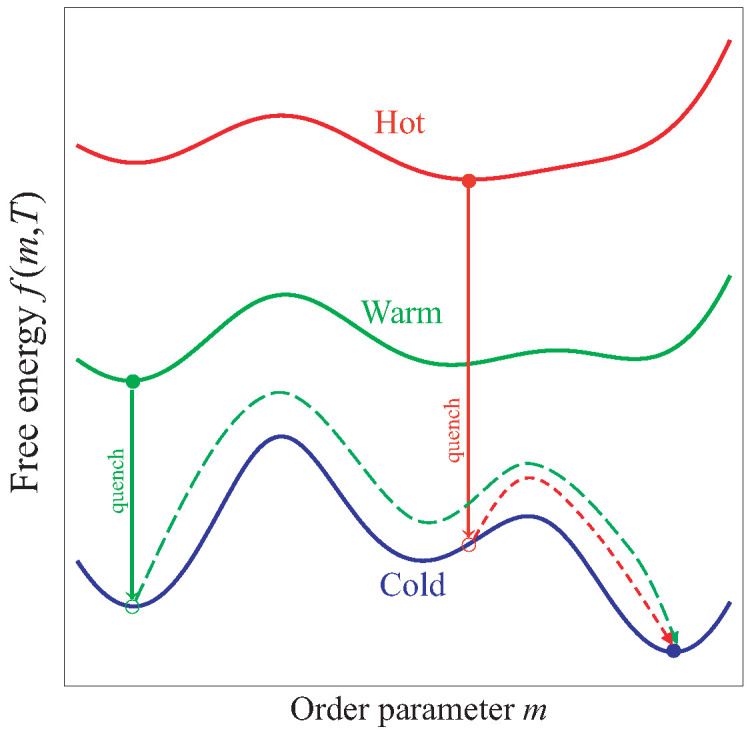
Schematic illustration of the proposed mechanism for the Mpemba effect. The free energy f(m,T) is plotted against the order parameter *m* for three different temperatures: High (Hot, red), Intermediate (Warm, green), and Low (Cold, blue). Filled circles represent the equilibrium states at each temperature. Upon quenching to the cold temperature, the systems are instantaneously projected onto the open circles on the blue (cold) landscape. The projection assumes the order parameter *m* is a slow variable during the instantaneous quench. The dashed arrows indicate their subsequent relaxation paths to the final cold equilibrium (blue filled circle). The system starting from the hot state (red) crosses one barrier, while the system starting from the warm state (green) must cross two barriers, leading to a shorter overall relaxation time for the hotter system.

In this section, we introduce a new theoretical mechanism that explains the emergence of the Mpemba effect in systems undergoing a first-order phase transition. The core idea is illustrated in Figure 1, which serves as the conceptual foundation of our work.

We consider a system whose thermodynamic state can be fully described by a single order parameter *m*. This simplification allows us to visualize the system’s free-energy landscape clearly. The free energy of the system, denoted as f(m,T), depends on both the order parameter *m* and the temperature *T*. The equilibrium state of the system at any given temperature corresponds to the global minimum of f(m,T).

Figure 1 depicts the free-energy curves at three different temperatures: a high “Hot” temperature (red curve), an intermediate “Warm” temperature (green curve), and a low “Cold” target temperature (blue curve). At the high temperature, the global minimum is located at an intermediate value of *m* (red filled circle). At the intermediate temperature, the equilibrium state shifts to a lower value of *m* (green filled circle). Finally, at the cold target temperature, the equilibrium state is found at a higher value of *m* (blue filled circle).

Now, consider two identical systems that differ only in their initial preparation. One system is prepared in equilibrium at the “Hot” temperature, and the other at the “Warm” temperature. Both are then simultaneously quenched into the same cold environment, whose temperature is fixed at $T_{\mathrm{cold}}$. Upon quenching, the free-energy landscape of the system instantaneously changes from its initial form to the one corresponding to $T_{\mathrm{cold}}$ (the blue curve). Crucially, the instantaneous value of the order parameter *m* does not change during this abrupt quench. This treatment is valid under the assumption that the quench is instantaneous on the timescale of the relaxation of the order parameter *m*, and that *m* constitutes a slow collective variable that separates from faster microscopic degrees of freedom. In a realistic finite-speed quench or non-Markovian setting, small deviations may occur, but the dominant relaxation path on the final free-energy landscape is expected to remain robust if the quench is sufficiently rapid.

As a result, the system that was initially “Hot” is projected onto the point marked by the red open circle on the cold landscape. Similarly, the initially “Warm” system is projected onto the green open circle. The subsequent relaxation dynamics are governed entirely by this new, cold free-energy landscape.

The key to the Mpemba effect lies in the distinct relaxation pathways these two systems must follow to reach the final equilibrium state (blue filled circle):

- **The Hot system:** Starting from the red open circle, which is centrally located, the system needs to cross only one free-energy barrier to reach the global minimum. Its path is direct and efficient.
- **The Warm system:** Starting from the green open circle, which lies deep within the basin of a metastable state (a local minimum), the system must first cross one barrier to enter this metastable state. It then can become trapped there for a prolonged period. To finally reach the true equilibrium, it must overcome a second, and often higher, free-energy barrier.

The time required to cross a free-energy barrier is dominated by the waiting time for a rare, large thermal fluctuation that provides sufficient energy to surmount the barrier. This waiting time is typically exponentially long and constitutes the vast majority of the total phase transition time. Since the initially hot system must overcome only one barrier, while the initially warm system must overcome two, the former will invariably reach the final low-temperature phase faster. This fundamental difference in the number of required barrier crossings provides a clear and general explanation for the Mpemba effect in the context of first-order phase transitions.

Of course, our description of the initially hot and initially warm systems crossing one and two energy barriers, respectively, is a simplification for clarity. The mechanism can be readily generalized to systems with more complex free-energy landscapes. For any system describable by a single order parameter, the Mpemba effect will arise whenever the relaxation path of the initially warmer system must overcome a greater number of free-energy barriers than that of the initially hotter system.

## 3. An Illustrative Example: The Extended Spin-1 Nagle–Kardar Model

### 3.1. The Extended Spin-1 Nagle–Kardar Model

To concretely demonstrate the mechanism proposed in Section 2, we now turn to a specific model system: the extended spin-1 Nagle–Kardar (NK) model. This model, defined on a one-dimensional chain of *N* spins where each spin variable $S_{i}$ can take the values −1,0,+1, is described by the Hamiltonian

(1) $$H=-\frac{J}{2N}{\left(\sum_{i=1}^{N}S_{i}\right)}^{2}-\frac{K}{2}\sum_{i=1}^{N}S_{i}S_{i+1}-\frac{\Delta }{2}\sum_{i=1}^{N}S_{i}^{2}S_{i+1}^{2}.$$

Here, the first term represents a mean-field ferromagnetic coupling of strength *J*, the second term is a nearest-neighbor bilinear interaction of strength *K*, and the third term is a nearest-neighbor biquadratic interaction of strength Δ. The competition between these interactions leads to a rich phase diagram [38,39].

For our analysis, we work in the canonical ensemble and set the long-range coupling J=1 without loss of generality. The thermodynamic properties of this model can be solved analytically using the transfer-matrix method combined with the Hubbard–Stratonovich transformation. The key outcome of this calculation is an explicit, analytical form for the free energy density f(T,m), which is a function of a single order parameter the magnetization per spin, m=〈S〉 and the temperature *T*.

**Figure 2:**
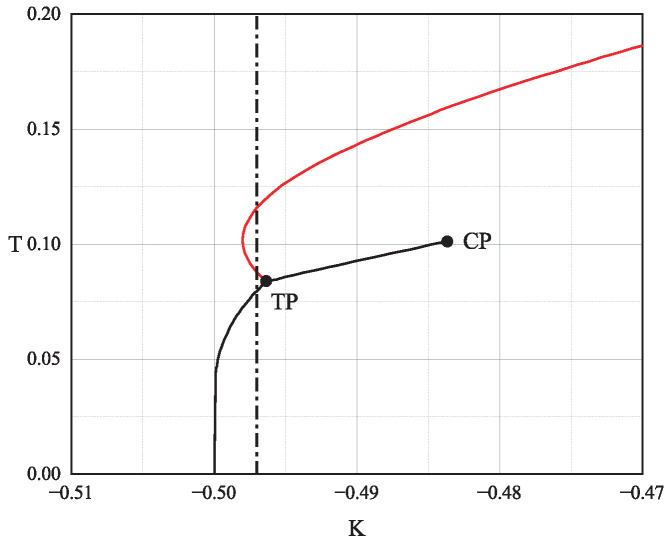
The canonical (K,T) phase diagram for the extended spin-1 Nagle–Kardar model with Δ=−0.23. Solid lines represent first-order phase transition lines. TP and CP denote the triple point and the critical point respectively. The vertical black dashed line at K=−0.497 marks the parameter value used for the subsequent analysis. This figure is adapted from Ref. [38].

To realize the scenario outlined in our schematic of Figure 1, we carefully select the model parameters as K=−0.497 and Δ=−0.23. These values lie within a distinctive region of the phase diagram where a triple point exists, allowing for a sequence of phase transitions as the temperature is varied [38]. Although these specific values are chosen to match the schematic in Figure 1, the underlying mechanism is not fine-tuned. The triple-point region in Figure 2 corresponds to a finite domain in parameter space where the coexistence of paramagnetic, stripe-ferromagnetic, and ferromagnetic phases enables the requisite free-energy topology for the Mpemba effect.

Figure 2 shows the canonical phase diagram in the (K,T) plane for the fixed value Δ=−0.23. This diagram, which is identical to Figure 4b of Ref. [38], features a triple point (TP) where three first-order phase transition lines meet. The solid lines denote first-order transitions. The black vertical dashed line at K=−0.497 indicates the specific parameter value we have chosen for our study. At this value of *K*, the system is poised to exhibit a complex sequence of phases as the temperature changes.

**Figure 3:**
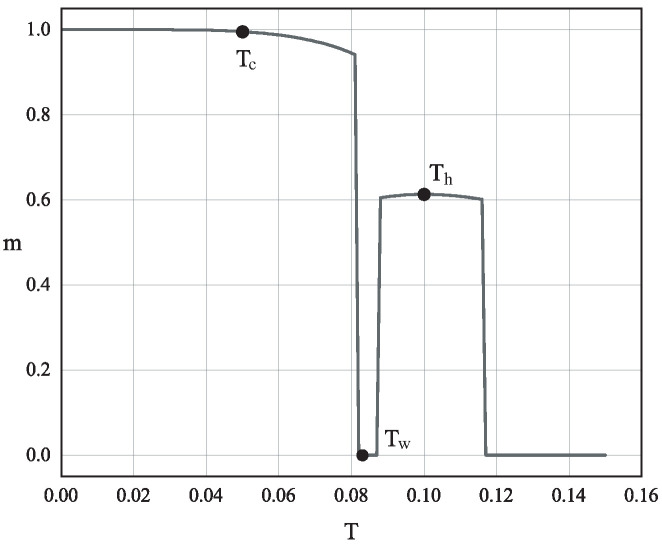
The equilibrium magnetization per spin *m* as a function of temperature *T* for the parameters K=−0.497 and Δ=−0.23. The three sharp jumps indicate first-order phase transitions. The three black dots mark the three temperatures used in our Mpemba effect study: the hot initial state ($T_{h}=0.100$), the warm initial state ($T_{w}=0.083$), and the cold final bath temperature ($T_{f}=0.050$). This figure is adapted from Ref. [38].

To understand the equilibrium states of the system at K=−0.497 and Δ=−0.23, we plot the magnetization per spin *m* as a function of temperature *T* in Figure 3. This curve, which replicates Figure 5a of Ref. [38], shows three distinct jumps in the magnetization, corresponding to three consecutive first-order phase transitions. Starting from a low temperature and heating the system, it transitions from a ferromagnetic state (m≈1) to a paramagnetic state (m=0), then to a stripe ferromagnetic state (m≈0.5), and finally back to the paramagnetic state (m=0).

**Figure 4:**
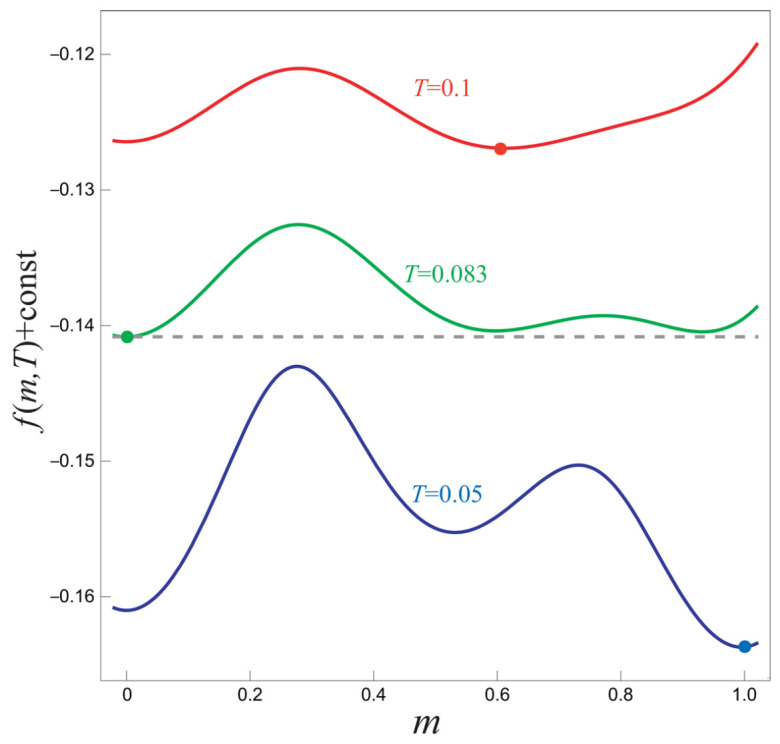
The analytic free energy density f(T,m) as a function of the order parameter *m* for the extended NK model with K=−0.497 and Δ=−0.23. The red, green, and blue curves correspond to the temperatures of the hot initial state ($T_{h}=0.100$), the warm initial state ($T_{w}=0.083$), and the final cold bath ($T_{c}=0.050$), respectively. The minima of these curves indicate the equilibrium states of the system at the respective temperatures, which map directly onto the states depicted in the schematic of Figure 1.

The equilibrium states at these three key temperatures are precisely what is needed to instantiate our theoretical mechanism. To visualize this, we plot the analytic free energy density f(T,m) for each of these temperatures in Figure 4. The red curve ($T_{h}=0.100$) shows that the equilibrium state for the hot system is at m≈0.5, which is the stripe ferromagnetic phase. The green curve ($T_{w}=0.083$) shows that the equilibrium state for the warm system is at m=0, the paramagnetic phase. Finally, the blue curve ($T_{c}=0.050$) shows that the true equilibrium state of the cold bath is at m≈1, the ferromagnetic phase.

This landscape perfectly mirrors the schematic in Figure 1. Upon a sudden quench to $T_{f}=0.050$, the free energy landscape instantly changes to the blue curve. The system that was initially hot (at m≈0.5) is projected onto the blue landscape at a point that is a direct neighbor of the global minimum at m≈1, requiring it to cross only a single free-energy barrier. In contrast, the system that was initially warm (at m=0) is projected onto a local minimum, a metastable state. To reach the final ferromagnetic equilibrium, it must first cross a barrier to escape this metastable well and then cross a second, lower barrier to enter the global minimum at m≈1. This difference in the number and height of the barriers to be crossed is the root cause of the Mpemba effect in this model.

### 3.2. Monte Carlo Simulation Protocol

To numerically investigate the relaxation dynamics of the extended spin-1 Nagle–Kardar model and to test the mechanism proposed in Section 2, we perform canonical Monte Carlo (MC) simulations. The simulation protocol is designed to mimic the quenching process central to the Mpemba effect.

The system consists of a one-dimensional chain of *N* spins, where each spin $S_{i}$ can take values in {−1,0,+1}. The Hamiltonian is given by Equation (1). Our goal is to prepare two identical systems in thermal equilibrium at two different initial temperatures, $T_{h}=0.100$ (the “hot” state) and $T_{w}=0.083$ (the “warm” state), and then to abruptly quench both to the same final cold bath temperature, $T_{f}=0.050$.

The simulation proceeds in two distinct stages. In the first stage, the system is equilibrated at its respective initial temperature. This is achieved by performing a sufficiently long MC run using the standard Metropolis algorithm with the temperature fixed at $T_{\mathrm{initial}}$. The acceptance probability for a proposed spin flip from an initial state with energy $E_{\mathrm{old}}$ to a new state with energy $E_{\mathrm{new}}$ is given by $\min\left[1,\exp\left(-\left(E_{\mathrm{new}}-E_{\mathrm{old}}\right)/T_{\mathrm{initial}}\right)\right]$. This stage ensures that the system samples its equilibrium configuration at $T_{\mathrm{initial}}$, which for $T_{h}$ corresponds to the stripe ferromagnetic state (m≈0.5) and for $T_{w}$ to the paramagnetic state (m=0), as shown in Figure 3.

At time t=0, the second stage begins: the thermal bath temperature is instantaneously switched from $T_{\mathrm{initial}}$ to the final temperature $T_{f}=0.050$. From this point onward, the MC dynamics proceeds using the Metropolis criterion with the new temperature $T_{f}$. The system, now far from equilibrium, begins its relaxation towards the final ferromagnetic ground state (m≈1).

The simulation is run until the system has definitively completed its phase transition. As shown in the free-energy landscape of Figure 4, the primary barrier to reaching the final equilibrium for a system starting from the warm state is the energy peak that separates the stripe ferromagnetic well (m≈0.5) from the ferromagnetic well (m≈1). Therefore, we define the phase transition as complete when the system’s magnetization per spin, *m*, first crosses the threshold value of m=0.95. At this point, the system is firmly within the basin of attraction of the final equilibrium and will quickly relax to m≈1. We have verified that our qualitative conclusion—the initially hot system reaches the final phase faster than the warm one—is robust against the choice of threshold; consistent results are obtained for m>0.9 and m>0.99.

For a given system size *N*, the relaxation time is quantified by the total number of MC steps required to reach this threshold after the quench at t=0. By comparing the average number of MC steps for the initially hot system versus the initially warm system, across multiple independent simulation runs, we can directly test for the presence of the Mpemba effect: if the hot system consistently reaches the final state in fewer MC steps than the warm system, the effect is verified. As the physical time is assumed to be proportional to the MC step count in this dynamics, this provides a robust measure of the relative phase transition times.

### 3.3. Monte Carlo Simulation Results

**Figure 5:**
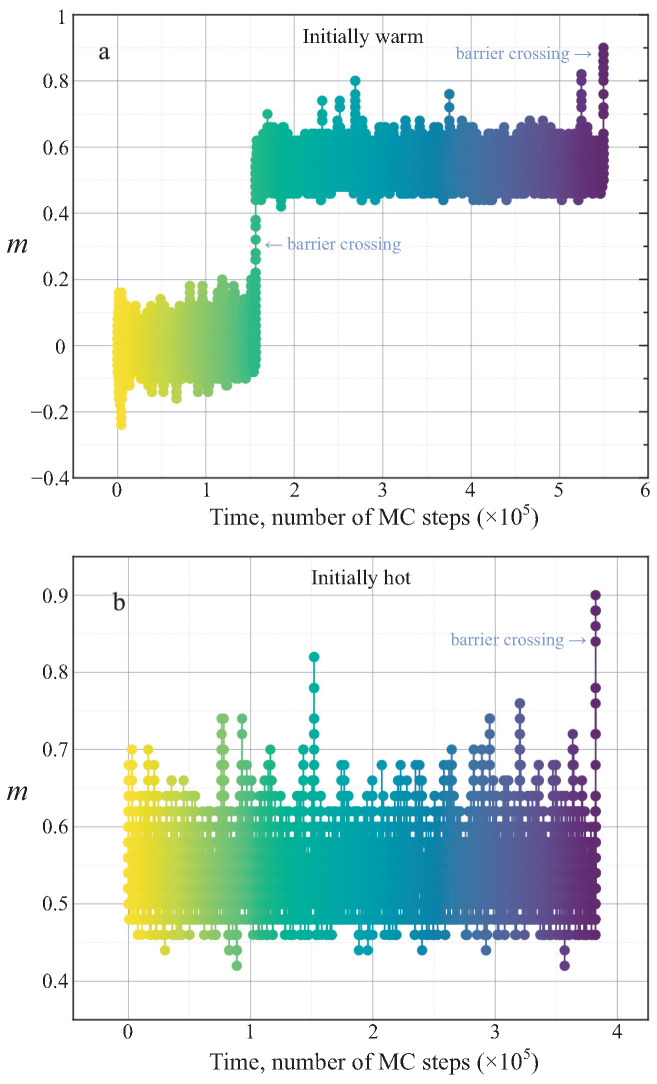
Evolution of the average magnetization *m* versus simulation time τ (number of MC steps) for a system with N=60 spins. Panel (**a**) corresponds to a system initialized at an intermediate temperature ($T_{w}=0.083$), while panel (**b**) corresponds to a system initialized at a higher temperature ($T_{h}=0.1$). The color gradient indicates the progression of time. In both panels, the horizontal plateaus correspond to the system being trapped in a metastable state within a potential well of the free energy landscape, while the sharp jumps signify the crossing of a free-energy barrier.

To illustrate the dynamics of the phase transition, we present the results from our MC simulations. Figure 5 displays the evolution of the average magnetization *m* as a function of simulation time τ, which is measured in MC steps, for a system with N=60 spins. This figure represents a single, independent simulation run.

As shown in Figure 5a, the system initialized at the intermediate temperature begins its evolution in the paramagnetic state, oscillating around m=0. After a significant period of thermal fluctuations, it successfully crosses a free-energy barrier and enters a new metastable state centered at m≈0.5. It remains trapped in this intermediate state for another prolonged duration before finally overcoming a second, smaller barrier to reach the final equilibrium ferromagnetic state at m≈1. In contrast, Figure 5b shows that the system initialized at the higher temperature starts directly in the m≈0.5 state, which is already close to the final equilibrium. Consequently, it only needs to cross a single barrier to complete the transition. This qualitative difference in the relaxation pathways explains why the total transition time for the initially hot system, denoted $\tau_{h}$, is shorter than that for the initially warm system, $\tau_{w}$.

**Figure 6:**
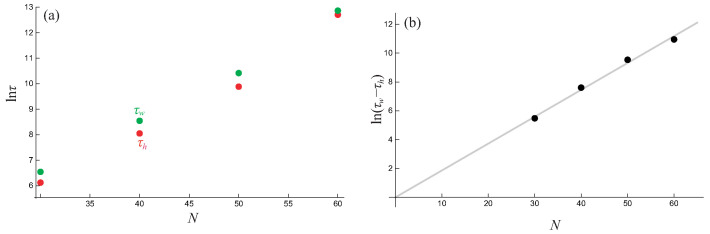
Analysis of the averaged transition time 〈τ〉 from 100 independent MC simulations. Panel (**a**) plots the natural logarithm of the mean transition time, ln〈τ〉, against the number of spins *N*. Red dots represent systems initialized at the high temperature ($T_{h}=0.1$), while green dots represent systems initialized at the intermediate temperature ($T_{w}=0.083$). Panel (**b**) plots the natural logarithm of the difference in transition times, $\ln(\langle \tau_{w}\rangle -\langle \tau_{h}\rangle )$, against *N*. The solid gray line is a linear fit to the data points, confirming the predicted exponential dependence on system size.

However, a single simulation is subject to large statistical fluctuations inherent in stochastic processes. To obtain statistically robust results, we performed 100 independent MC simulations for each system size (N=30,40,50,60). For each system size, we calculated the mean transition time, 〈τ〉, by averaging over these 100 runs. The results are presented in Figure 6.

Figure 6a clearly demonstrates that for all system sizes studied, the mean evolution time for the initially hot system is consistently shorter than for the initially warm system. This robustly confirms the occurrence of the Mpemba effect in our model. The difference in transition times, $\langle \tau_{w}\rangle -\langle \tau_{h}\rangle$, arises because the initially warm system must spend additional time crossing the first barrier to escape the initial paramagnetic state (m=0). According to the Arrhenius law, the characteristic time for crossing a barrier is proportional to $\exp(\Delta F/k_{B}T)$, where ΔF is the free-energy barrier height. In our mean-field model, the barrier height scales with the system size as ΔF=N·δf, where δf is a constant free-energy density difference. Therefore, the time difference should scale exponentially with *N*, implying that $\ln(\langle \tau_{w}\rangle -\langle \tau_{h}\rangle )$ should be linearly proportional to *N*. This prediction is confirmed in Figure 6b, where the data points for $\ln(\langle \tau_{w}\rangle -\langle \tau_{h}\rangle )$ lie approximately on a straight line passing through the origin, validating our theoretical interpretation.

## 4. Conclusions

In this work, we have presented a novel and minimal mechanism for the genuine Mpemba effect—a phenomenon that occurs in the context of a first-order phase transition, where the initially hot system enters the low-temperature phase faster than an initially warm one. Our mechanism hinges on a simple yet powerful geometric picture in the order parameter space: the relaxation pathway following a sudden quench is determined by the location of the initial equilibrium state relative to the free-energy landscape of the final cold phase. We have demonstrated that if the hot initial state is projected onto a region of the landscape that requires the system to overcome fewer free-energy barriers than the warm initial state, the counterintuitive acceleration of the phase transition is a natural consequence.

This mechanism is distinct from previous proposals in two crucial aspects. First, it is remarkably parsimonious, requiring only a single order parameter to fully describe the system’s state and its associated free-energy landscape. This stands in contrast to other theoretical models—for instance, the Blume–Emery–Griffiths model—which rely on at least two coupled order parameters to create the necessary topological separation between basins of attraction [36,37]. Our work shows that such complexity is not a prerequisite for the effect. The exponential scaling of the time difference with system size (Figure 6b), characteristic of activated barrier-hopping dynamics, strongly supports a geometric interpretation based on the topology of the free-energy landscape as the most direct and intuitive explanation for the Mpemba effect in this setting. While mode projection-based frameworks [29] may provide complementary perspectives in certain contexts, the observed relaxation pathway is most naturally understood in terms of the number and sequence of free-energy barriers that must be traversed.

Second, our framework operates entirely within the canonical, Markovian paradigm of stochastic thermodynamics. The effect arises purely from the structure of the equilibrium free energy and the ensuing non-equilibrium relaxation dynamics, without any need to invoke a finite-sized, back-reacting thermal reservoir or other non-Markovian elements [24,34,35]. This distinguishes our mechanism from the non-Markovian Mpemba effect previously identified in mean-field systems and underscores that the phenomenon can emerge generically in any system whose free-energy landscape features the requisite arrangement of metastable and stable states.

By providing a clear, general, and minimal explanation rooted in the fundamental concepts of phase transitions and barrier-crossing kinetics, our work offers a new perspective on the century-old Mpemba puzzle. It suggests that the key to understanding why “hotter can be faster” in systems undergoing freezing or other first-order transitions may lie not in the peculiarities of water or non-equilibrium reservoir physics, but in the universal geometric properties of their underlying free-energy landscapes.

## Data Availability

The original contributions presented in this study are included in the article. Further inquiries can be directed to the corresponding author.
